# Fecal carriage of extended-spectrum beta-lactamase-producing Enterobacterales in healthy Spanish schoolchildren

**DOI:** 10.3389/fmicb.2023.1035291

**Published:** 2023-06-09

**Authors:** Mireia López-Siles, Zaira Moure, Aly Salimo Muadica, Sergio Sánchez, Raquel Cruces, Alicia Ávila, Noelia Lara, Pamela Carolina Köster, Alejandro Dashti, Jesús Oteo-Iglesias, David Carmena, Michael J. McConnell

**Affiliations:** ^1^Intrahospital Infections Unit, Reference and Research Laboratory in Resistance to Antibiotics and Infections Related to Healthcare, National Centre for Microbiology, Instituto de Salud Carlos III (ISCIII), Madrid, Spain; ^2^Serra Húnter Fellow, Microbiology of Intestinal Diseases, Biology Department, Universitat de Girona, Girona, Spain; ^3^Reference and Research Laboratory in Resistance to Antibiotics and Infections Related to Healthcare, National Centre for Microbiology, Instituto de Salud Carlos III, Madrid, Spain; ^4^Servicio de Microbiología, Hospital Universitario Marqués de Valdecilla, Instituto de Investigación Sanitaria, (IDIVAL), Santander, Spain; ^5^Parasitology Reference and Research Laboratory, National Centre for Microbiology, Instituto de Salud Carlos III, Madrid, Spain; ^6^Departamento de Ciências e Tecnologia, Universidade Licungo, Quelimane, Mozambique; ^7^Reference and Research Laboratory of Food and Waterborne Bacterial Infections, National Center for Microbiology, Instituto de Salud Carlos III, Madrid, Spain; ^8^Spanish Network for Research in Infectious Diseases (REIPI RD16/0016), Instituto de Salud Carlos III, Madrid, Spain; ^9^Centro de Investigación Biomédica en Red de Enfermedades Infecciosas (CIBERINFEC), Instituto de Salud Carlos III, Madrid, Spain

**Keywords:** extended-spectrum ß-lactamase (ESBL), multidrug resistance (MDR) bacteria, Enterobacterales, fecal carriage, children

## Abstract

**Background:**

Extended-spectrum ß-lactamase-producing Enterobacterales (ESBL-E) are a serious threat among emerging antibiotic resistant bacteria. Particularly, the number of cases of ESBL-E infections reported in children has been increasing in recent years, and approved antibiotic treatments for this age group are limited. However, information regarding the prevalence of colonization in European children, risk factors associated with colonization, and the characteristics of the colonizing strains is scarce. The aims of this study were to determine the prevalence of ESBL-E colonization in fecal samples of apparently healthy schoolchildren, to identify lifestyle routines associated with colonization, and to characterize clonal relationships and mechanisms of resistance in ESBL-E isolates.

**Methods:**

A cohort of 887 healthy children (3–13  years old) from seven primary and secondary schools in the Madrid metropolitan area was recruited between April–June 2018, and sociodemographic information and daily habits were collected. Fecal samples were screened for ESBL-E carriage in selective medium. ESBL-E isolates were further characterized by assessing molecular epidemiology (PFGE and MLST), ESBL gene carriage, and antibiotic resistance profile. This information was analyzed in conjunction with the metadata of the participants in order to identify external factors associated with ESBL-E carriage.

**Results:**

Twenty four ESBL-E, all but one *Escherichia coli,* were detected in 23 children (prevalence: 2.6%; 95% CI: 1.6–3.6%). Of these, seven contained the *bla*_CTX-M-14_ allele, five the *bla*_CTX-M-15_, five the *bla*_SHV-12_, three the *bla*_CTX-M-27_, three the *bla*_CTX-M-32_, and one the *bla*_CTX-M-9_. Significant clonal diversity was observed among the isolates that grouped into 22 distinct clusters (at <85% similarity of PFGE profile). ESBL-producing *E. coli* isolates belonged to 12 different STs, with ST10 (25%) and ST131 (17%) being the most frequent. Apart from ß-lactams, resistance to trimethoprim/sulfamethoxazole (46%), ciprofloxacin (33%), levofloxacin (33%), tobramycin (21%), and gentamicin (8%) were the most frequently detected.

**Conclusion:**

The prevalence of ESBL-E in the studied cohort of children was lower than the average colonization rate previously detected in Europe for both children and adults. *E. coli* was the main ESBL-producing species detected and CTX-M were the most frequently identified ESBLs. High ST diversity suggests polyclonal dissemination. Compared to other STs, ST131 isolates were associated with resistance to various antimicrobials.

## 1. Introduction

The global dissemination of bacterial strains with resistance to antibiotics represents an important Public Health challenge (European Centre for Disease Prevention and Control, 2019). Particularly, extended spectrum ß-lactamase-producing Enterobacterales (ESBL-E) are considered a serious threat as they are resistant to one of the most frequently used families of antibiotics (ß-lactams, including cephalosporins). It has been estimated that, including both hospital- and community-onset, in 2019 there were at least 197,400 infections and 9,100 deaths caused by this group of drug-resistant bacteria in the United States alone according to the Centers for Disease Control and Prevention (CDC, 2019). Additionally, ESBL-E often carry genes encoding resistance to other antimicrobial groups such as aminoglycosides, fluoroquinolones, tetracycline, and trimethoprim-sulfamethoxazole (Coque et al., 2008; Pitout and Laupland, 2008; Guimarães et al., 2009; Rodríguez-Revuelta et al., 2018), therefore reducing the number of treatment options available. This is of particular concern in children, when treatment options are reduced, and is aggravated by the fact that ESBL-E are on the rise as causes of infections in this age group (Lukac et al., 2015).

A recent review and meta-analysis evidenced an 8-fold increase in the intestinal carriage rate of ESBL-*Escherichia coli* in the community over the past two decades, with most recent prevalence values reaching 21.1% (Bezabih et al., 2021). In Spanish cohorts, in the past decade it was established that ESBL-E carriage prevalence ranged from 3.7 to 7.4% in non-hospitalized adults (Valverde et al., 2004; Rodríguez-Baño et al., 2008; Vinué et al., 2009) and has increased since the 1990s (Valverde et al., 2004; Romero et al., 2005). However, there is little information regarding ESBL-E carriage in healthy Spanish children. In contrast, some studies have been performed engaging hospitalized Spanish children, and evidenced that 24% of children aged from 8 to 16-months old are ESBL-E carriers, which was among the highest prevalence data reported in Europe at the time of study (Fernández-Reyes et al., 2014). Therefore, information regarding carriage rates in the community within this age group would be of interest.

Some studies have explored factors related to colonization in children. Having a colonized mother was reported to increase risk of colonization (Rodríguez-Revuelta et al., 2018). A study carried out in China evidenced that lower space per person among households may play a role in ESBL-E colonization (Lo et al., 2010). Gender and having two or more siblings was not associated with ESBL-E colonization (Fernández-Reyes et al., 2014). Sterilization of feeding bottles was protective for ESBL-E acquisition (Rodríguez-Revuelta et al., 2018). Out-of-home child-care was demonstrated to influence ESBL-E carriage, but differing results were reported between studies (Valverde et al., 2008; Kaarme et al., 2013; Fernández-Reyes et al., 2014; Rodríguez-Revuelta et al., 2018). Varied results were also found for living with pets and breastfeeding as in some studies it was shown as protective for ESBL-E acquisition (Rodríguez-Revuelta et al., 2018) whereas in others no association with ESBL-E colonization was found (Fernández-Reyes et al., 2014). Lastly, regarding antibiotic consumption, a study associated a higher risk of community carriage of ESBL in French children (aged 6–24 month) to previous oral intake of third-generation cephalosporins (Birgy et al., 2012) whereas previous antibiotic therapy (during the first 16 months of life, or 4 months before sampling) was not associated with ESBL-E colonization in a different study engaging Spanish children (Fernández-Reyes et al., 2014).

In this context, we determined the prevalence of ESBL-E fecal carriage in school-age Spanish children in the community setting, analyzed the molecular epidemiology of ESBL-E isolates, and characterized them in terms of resistance gene content and antimicrobial susceptibility profile. In addition, we analyzed these data in conjunction with the sociodemographic variables of the cohort in order to identify external risk/preventive factors associated with ESBL-E carriage.

## 2. Materials and methods

### 2.1. Volunteer recruitment, sample collection, and metadata

**Figure 1:**
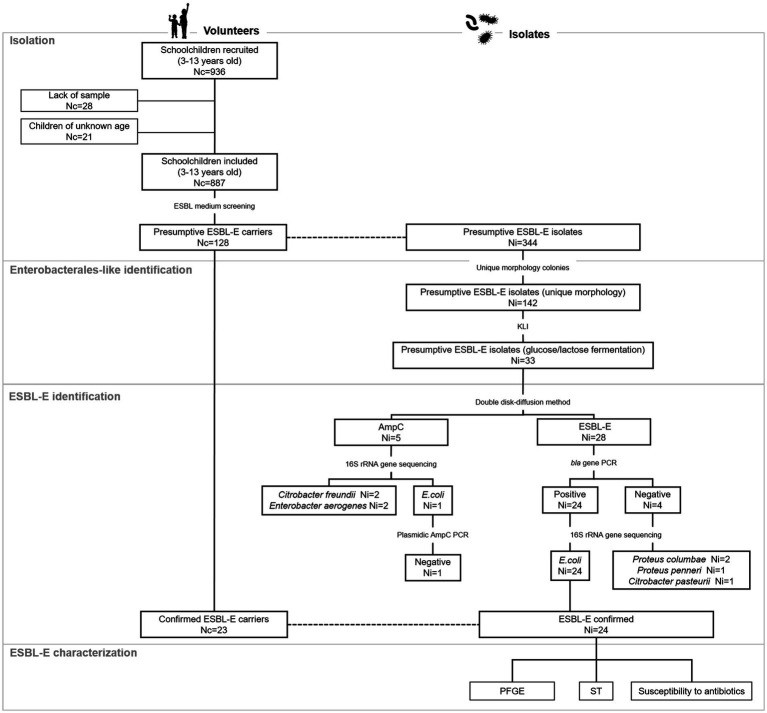
Flow diagram of the study. Nc, number of children; Ni, number of isolates; ESBL-E, Extended-spectrum ß-lactamase-producing Enterobacterales; PCR, polymerase chain reaction; PFGE, Pulsed-field gel electrophoresis; ST, sequence type.

A retrospective cross-sectional study was conducted on a cohort from a previous study (Reh et al., 2019) that engaged 936 children attending seven public primary and secondary schools in the southern metropolitan area of Madrid. Stool samples were collected between April and June 2018, with the assistance of parents/legal guardians of children. They were previously trained through informative meetings and a sampling kit (sterile polystyrene plastic flask with spatula and a unique identification number) was provided for this purpose. Fecal samples were brought to school at room temperature within the following 24 h after deposition, and then transported to the Spanish National Centre for Microbiology and stored at −80°C without preservatives until further analyses. Because in some cases key data or sufficient sample volume were not provided, 887 out of 936 fecal samples were analyzed (Figure 1).

Metadata for each sample, obtained in the previous study through a standardized questionnaire (Reh et al., 2019) were provided (Table 1). Information included: (i) demographic characteristics, e.g., age, sex, number of siblings, school, and WHO region of origin (ii) behavioral habits, e.g., fruit/vegetable washing and recent history of diarrheal episodes in the participant and/or their family members, and (iii) additional information on potential risk/protective factors for protozoan enteroparasites colonization also relevant in ESBL-E colonization, e.g., types of drinking water, if there has been any contact with pets and any recent travel abroad. Information regarding enteric protist presence/absence, including *Cryptosporidium* spp., *Giardia duodenalis*, and *Blastocystis* sp. established through molecular methods (Reh et al., 2019), was also provided. In addition, consistency of feces according to Bristol stool chart was recorded and considered in the analysis (Lewis and Heaton, 1997).

**Table 1:** Cohort of subjects and characteristics by extended spectrum ß-lactamase-producing Enterobacterales (ESBL-E) carriage condition.

		n^a^		All subjects	ESBL-E non-carriers	ESBL-E carriers	*p*-value^b^	Test^b^
n^a^				887	864	23		
Age		887	Mean years ± SD	7.3 ± 2.7	7.3 ± 2.7	6.6 ± 2.3	0.219	¥
Gender		887	n males (%)	507 (57%)	493 (57%)	14 (61%)	0.832	#
School		887	n (%)				0.796	#
	Ángel González			233 (26%)	227 (26%)	6 (26%)		
	Antanes School			133 (15%)	128 (15%)	5 (27%)		
	Carmen Conde			124 (14%)	122 (14%)	2 (8.7%)		
	León Felipe			71 (8.0%)	69 (8.0%)	2 (8.7%)		
	Lope de Vega			136 (15%)	132 (15%)	4 (17%)		
	Manuel Vázquez Montalbán			127 (14%)	123 (14%)	4 (17%)		
	Pequeño Príncipe			63 (7.1%)	63 (7.3%)	0 (0.0%)		
Bristol scale		701	n (%)				0.438	#
	1			50 (7.1%)	50 (7.4%)	0 (0.0%)		
	2			97 (14%)	94 (14%)	3 (14%)		
	3			81 (12%)	79 (12%)	2 (9.5%)		
	4			150 (21%)	144 (21%)	6 (29%)		
	5			147 (21%)	140 (21%)	7 (33%)		
	6			107 (15%)	104 (15%)	3 (14%)		
	7			69 (9.8%)	69 (10%)	0 (0.0%)		
WHO region of origin		870	n subjects (%)				0.643	#
	America			52 (6.0%)	50 (5.9%)	2 (8.7%)		
	Europe			818 (94%)	797 (94%)	21 (91%)		
Number of siblings		866	n subjects (%)					
	None			172 (20%)	167 (20%)	5 (24%)	0.973	#
	1			583 (67%)	569 (67%)	14 (67%)		
	2			101 (12%)	99 (12%)	2 (9.5%)		
	≥3			10 (1.1%)	9 (1.1%)	0 (0.0%)		
Diarrhea the past 7 days		883	n subjects (%)	31 (3.5%)	30 (3.5%)	1 (4.3%)	0.565	#
Family member with diarrhea the past 7 days		876		112 (13%)	108 (13%)	4 (17%)	0.522	#
Pets’ owner		885	n subjects (%)				0.405	#
	No pets			688 (78%)	671 (78%)	17 (74%)		
	Cats			51 (5.8%)	48 (5.6%)	3 (13%)		
	Dogs			120 (14%)	117 (14%)	3 (13%)		
	Cats&Dogs			26 (2.9%)	26 (3.0%)	0 (0.0%)		
Source of drinking water		884	n subjects (%)				0.864	#
	Tap water			641 (73%)	626 (73%)	15 (65%)		
	Bottled water			53 (6.0%)	52 (6.0%)	1 (4.3%)		
	Tap Water&Bottled water			124 (14%)	119 (14%)	5 (22%)		
	Tap Water&Bottled water&spring/fountains			12 (1.4%)	12 (1.4%)	0 (0.0%)		
	Tap Water&spring/fountains			54 (6.1%)	52 (6.0%)	2 (8.7%)		
Vegetables washing		880	n subjects (%)				0.217	#
	Always			645 (73%)	631 (74%)	14 (61%)		
	Habitually			216 (25%)	207 (24%)	9 (39%)		
	Rarely			19 (2.2%)	19 (2.2%)	0 (0.0%)		
Travel abroad in the past 6 months		881	n subjects (%)	162 (18%)	159 (19%)	3 (14%)	0.782	#
Travel to EU in the past 6 months		162	n subjects (%)	58 (36%)	56 (35%)	2 (67%)	0.292	#
Enteric protozoa		887	n carriers (%)	292 (33%)	283 (33%)	9 (39%)	0.508	#

^a^Number of subjects out of the 887 participant that provided information for each variable is indicated.

^b^*p*-value refers to comparison between ESBL-E carriers and non-carriers; ¥ *t*-test, # Chi-square test. Data had a normal distribution (Kolmogorov–Smirnov test, *p* = 0.511) and variances were equal between groups (Levene’s statistics, *p* = 0.153).

### 2.2. ESBL-E isolation and identification

To screen for ESBL-E, a 10 μL loop of fecal material was resuspended in 500 μL of phosphate-buffered saline (PBS), vigorously vortexed, and 100 μL of this suspension were spread onto chromID ESBL agar (bioMérieux, Marcy-l’Étoile, France). After overnight growth at 37°C, up to three colonies per plate, with different morphotypes and compatible with ESBL-E phenotype were re-isolated onto ESBL agar. Colonies confirmed to be able to grow on selective ESBL medium were further spread onto Luria-Bertani plates to re-assess for purity and different morphotypes.

Up to two isolates per subject were inoculated into Kligler Iron Agar medium (bioMérieux) to assess glucose and lactose fermentation and H_2_S production. In addition, catalase enzyme presence was tested by exposure to H_2_O_2_.

Finally, colonies with characteristics compatible with Enterobacterales were identified using 16S rRNA gene sequencing. Briefly, genomic DNA was obtained by freeze-and-thaw and PCR was performed using the universal bacterial primers 7F and 1510R as described previously (Lopez-Siles et al., 2012; Supplementary Table 1). PCR products were purified with the NucleoSpin^®^ Gel and PCR Clean-up kit (Macherey-Nagel, Dueren, Germany) according to manufacturer recommendations. Bidirectional partial 16S rRNA gene sequences were obtained by using the same primers on a ABI3730XL Capillary Electrophoresis Sequencing System (Applied Biosystems, Waltham, MA, United States). All primers were obtained with desalted purification (Sigma, Darmstadt, Germany).

### 2.3. ESBL-E confirmation, gene variant identification, and antibiotic resistance profile

Confirmed Enterobacterales isolates were studied for ESBL and/or AmpC type enzymes as previously reported (González-Torralba et al., 2016). The phenotypic confirmation method used was the combination disk test, which included a disk containing cefotaxime (30 μg) alone and in combination with clavulanate, cloxacillin, and cloxacillin-clavulanate (Ref. 98,008, Rosco, Taastrup, Denmark). The presence of genes encoding ESBLs (*bla*_SHV_, *bla*_CTX-M-1_, and *bla*_CTX-M-9_ groups) was determined by use of PCR and subsequent DNA sequencing (Supplementary Table 1). The sequences obtained were compared with those available in the GenBank public repository.

Antimicrobial susceptibility to 32 clinically relevant antibiotics was determined by microdilution method using the MicroScan (NM52) panel following the manufacturer’s recommendations (Beckman Coulter, L’Hospitalet de Llobregat, Spain). EUCAST guidelines at the time of study were used for interpretation of susceptibility/resistance breakpoints in all cases except for cefoxitin, where CLSI guideline was used for interpretation (Clinical and Laboratory Standards Institute, 2020; The European Committee on Antimicrobial Susceptibility Testing, 2021).

### 2.4. Molecular epidemiology

Pulsed-field gel electrophoresis (PFGE) analysis with *XbaI* restriction enzyme digestion was used to establish relatedness among isolates in accordance with the PulseNet standard protocol.^1^,^2^ Indistinguishable *Xba*I profiles were confirmed by a second analysis with *Bln*I digestion. The resulting PFGE profiles were analyzed with the InfoQuestFP software version 4.5 (Bio-Rad, Hemel Hempstead, United Kingdom). Cluster analysis was performed using the Dice coefficient and the unweighted pair group method with arithmetic averages (UPGMA).

Multiple locus sequence typing (MLST) was performed according to the Enterobase scheme (Wirth et al., 2006; Supplementary Table 1). Sequences of the *adk*, *fumC*, *gyrB*, *icd*, *mdh*, *purA*, and *recA* genes were compared to those in the PubMLST to assign sequence types (ST) (Jolley et al., 2018).

### 2.5. Statistics

Normal distribution of data was assessed using the Kolmogorov–Smirnov test, and Levene’s statistics was used to assess the equality of variances for groups. For continuous variables, the *t*-test was used to assess differences in mean between ESBL-E carriers and non-carriers. The Chi square test was used for categorical variables, in order to reveal differences in ESBL-E prevalence related to sociodemographic variables, clonal complex, ST, *bla* gene distribution, and antibiotic resistance profiles. Frequency distribution of sociodemographic variables was analyzed only if all categories included at least two observations. All statistical analyses were carried out in IBM SPSS statistics version 15.0. Statistical significance was set at *p* ≤ 0.05.

## 3. Results

### 3.1. ESBL-E screening, isolation, and confirmation

Initially, 936 children were recruited into the study. Of those, 28 were excluded because not enough sample was provided and 21 were discarded because of missing key data (age); therefore, 887 children aged 3–13 years old (median ± SD: 7.0 ± 2.7 years old; male/female ratio: 507/380) were included for ESBL-E screening. Main features of the study participants are shown in Table 1.

A flow chart summarizing the isolation procedure and ESBL-E screening is depicted in Figure 1. After initial inoculation on ESBL medium, growth was detected in 128 out of 887 samples analyzed (14%). Up to three colonies per individual were selected to assess purity and colony morphotype; thus, 344 isolates were assessed. A collection of 142 isolates likely to produce ESBL from 94 participants, with unique colony morphology (1 or 2 per individual), were selected for further Enterobacterales-like phenotype confirmation. From those, 33 isolates were either glucose or lactose fermenters and thus, regarded as presumptive ESBL-E. Combination disk test revealed that 28 isolates had a profile consistent with ESBL production, whereas five had a profile indicating AmpC production. From the former, 24 isolates from 23 subjects were confirmed as *bla*-containing isolates by PCR. Sequencing at the 16S rRNA gene revealed that all isolates but one belonged to *Escherichia coli* species. The remaining isolate (Isolate 5) was *Escherichia marmotae.* No *bla* genes were detected by molecular methods in 4 isolates with ESBL-like profile and they were identified as either *Proteus columbae, Proteus penneri* or *Citrobacter pasteurii.* Regarding isolates with AmpC profile, one isolate was *E. coli* but plasmidic AmpC was not confirmed by molecular methods. The remaining four isolates with AmpC profile were identified as *Citrobacter freundii* or *Enterobacter aerogenes*.

### 3.2. ESBL-E prevalence values and relation to sociodemographic data

The presence of ESBL-E was confirmed in 23 out of 887 children screened (Table 1). Therefore, the overall prevalence of colonization was 2.6% (95% CI: 1.6–3.6%). After morphotype screening, a single ESBL-E isolate remained per subject, except for one child, who carried two isolates with different morphotypes. Thus, the mean ESBL-E carriage rate detected by our approach was 1.04 ± 0.21 isolates/colonized individual.

Distribution of ESBL-E carriers was analyzed taking into account the demographic characteristics of children, the behavioral habits reported by parents/legal guardians and information concerning risk/protective factors for colonization (Table 1). None of these factors, correlated with prevalence of ESBL-E carriage.

### 3.3. Molecular epidemiology of ESBL-E isolates

**Figure 2:**
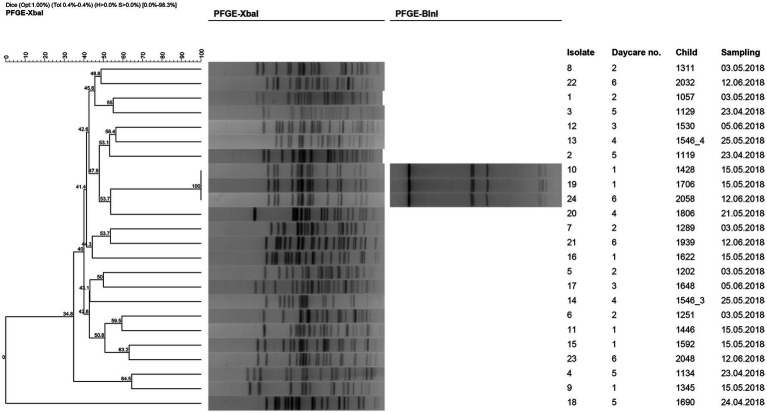
Consensus UPGMA dendrogram with a 0.4 tolerance value generated from Dice coefficients of *Xba*I pulsed field gel electrophoresis profiles of the 24 ESBL-E isolates. Numbers at branching points indicate % of similarity between profiles. Profiles with at least one different band were considered different pulsotypes. For those isolates with indistinguishable pulsotype after digestion with *Xba*I, restriction profile of *Bln*I is also shown. Isolation information is listed including children ID, sampling date and daycare (1, Ángel González; 2, Antanes School; 3, Carmen Conde; 4, León Felipe; 5, Lope de Vega; 6, Manuel Vázquez Montalbán).

In total, 22 clonally distinct PFGE profiles (<85% similarity) of 24 isolates from 23 children were identified (Figure 2). Three isolates from three different children attending to two different schools showed profiles that were identical.

The clonal complex and MLST designations of the isolates were determined to assess their genetic relatedness (Table 2). With regard to ST, while ST10 (25%) and ST131 (17%) were the most dominant; the majority of the isolates belonged to a unique ST. Overall, isolates were distributed between 10 different STs. Isolates belonged to six different clonal complexes. Three STs were not assigned to a clonal complex. The most frequent clonal complexes were ST10 Cplx (33%) and ST131 Cplx (17%). The two isolates (Isolates 13 and 14) obtained from the same subject were from a different ST, clonal complex and had different PFGE profile which confirms that are a different ESBL-E strain.

**Table 2:** Age and gender of children of isolation source, sequence type, type of extended spectrum ß-lactamase (ESBL) enzymes and antimicrobial resistance phenotype of 24 ESBL-E isolates.

							ESBL		Antimicrobials resistance^b^
Isolate	ID child	Child age (years)	Child gender	Daycare n°^a^	Clonal complex	ST	Enzyme group	Enzyme type	Beta-lactams	Quinolones	Others
1	1,057	6	F	2	ST10 Cplx	48	CTX-M-9	CTX-M-9	AMP, TIC, P, MEC, CRM, CFE, FOT, CPD, *CAZ*, FEP, AZT	NXN, CIP, LEV	GEN, TOB, TRI, TRS
2	1,119	9	M	5	ST155 Cplx	58	CTX-M-9	CTX-M-14	AMP, TIC, P, CRM, CFE, FOT, CPD, FEP, AZT		TRI, TRS
3	1,129	5	M	5	ST40 Cplx	40	CTX-M-9	CTX-M-14	AMP, TIC, P, AMC, CRM, CFE, FOT, CPD, FEP, AZT		
4	1,134	8	M	5	ST69 Cplx	69	CTX-M-1	CTX-M-15	AMP, TIC, P, CRM, CFE, FOT, CPD, CAZ, FEP, AZT		TRI, TRS
5	1,202	8	M	2	None^c^	None ^c^	SHV	SHV-12	AMP, TIC, P, CRM, CFE, FOT, CPD, CAZ, FEP, AZT		
6	1,251	8	M	2	ST10 Cplx	10	SHV	SHV-12	AMP, TIC, P, CFE, FOT, CPD, CAZ, AZT		TRI, TRS
7	1,289	5	F	2	ST131 Cplx	131	CTX-M-9	CTX-M-27	AMP, TIC, P, CRM, CFE, FOT, CPD, CAZ, FEP, AZT	NXN	GEN, TOB, NIT, TRI, TRS
8	1,311	10	F	2	ST155 Cplx	58	CTX-M-9	CTX-M-14	AMP, TIC, P, CRM, CFE, FOT, CPD, FEP, AZT		
9	1,345	6	F	1	ST69 Cplx	69	CTX-M-1	CTX-M-15	AMP, TIC, P, CRM, CFE, FOT, CPD, CAZ, FEP, AZT		
10	1,428	8	M	1	ST10 Cplx	10	CTX-M-1	CTX-M-32	AMP, TIC, P, CRM, CFE, FOT, CPD, CAZ, FEP, AZT		
11	1,446	3	M	1	None	1,684	CTX-M-9	CTX-M-14	AMP, TIC, P, CRM, CFE, FOT, CPD, FEP, AZT		AK
12	1,530	8	F	3	ST155 Cplx	58	CTX-M-9	CTX-M-14	AMP, TIC, P, CRM, FOT, CPD, *FEP, AZT*		TRI, TRS
13	1,546	3	M	4	ST10 Cplx	10	SHV	SHV-12	AMP, TIC, P, CRM, CFE, FOT, CPD, CAZ, FEP, AZT	NXN, CIP, LEV	
14	1,546	3	M	4	None	164	SHV	SHV-12	AMP, TIC, P, CRM, CFE, FOT, CPD, CAZ, AZT		
15	1,592	10	M	1	ST23 Cplx	90	SHV	SHV-12	AMP, TIC, P, FOT, CPD, *AZT*	NXN, CIP, LEV	
16	1,622	11	M	1	ST131 Cplx	131	CTX-M-1	CTX-M-15	AMP, TIC, P, AMC, CRM, CFE, FOT, CPD, AZT	NXN, CIP, LEV	TOB, TRI, TRS
17	1,648	4	M	3	None	752	CTX-M-9	CTX-M-14	AMP, TIC, P, CRM, CFE, FOT, CPD, FEP, AZT	NXN	
18	1,690	3	M	5	ST131 Cplx	131	CTX-M-1	CTX-M-15	AMP, TIC, P, AMC, CRM, CFE, FOT, CPD, *CAZ, AZT*	NXN, CIP, LEV	AK, TOB, TRI, TRS
19	1706	5	F	1	ST10 Cplx	10	CTX-M-1	CTX-M-32	AMP, TIC, P, CRM, CFE, FOT, CPD, CAZ, FEP, AZT		
20	1806	6	M	4	ST10 Cplx	617	CTX-M-1	CTX-M-15	AMP, TIC, P, CRM, CFE, FOT, CPD, CAZ, FEP, AZT	NXN, CIP, LEV	TOB, TRI, TRS
21	1939	7	F	6	ST131 Cplx	131	CTX-M-9	CTX-M-27	AMP, TIC, P, *FOX,* CRM, CFE, FOT, CPD, CAZ, FEP, AZT	NXN, CIP, LEV	TRI, TRS
22	2032	7	F	6	ST10 Cplx	10	CTX-M-9	CTX-M-14	AMP, TIC, P, CRM, CFE, FOT, CPD, FEP, *AZT*		
23	2048	6	M	6	ST23 Cplx	90	CTX-M-9	CTX-M-27	AMP, TIC, P, CRM, CFE, FOT, CPD, *CAZ, FEP,* AZT	NXN, CIP, LEV	TRI, TRS
24	2058	6	F	6	ST10 Cplx	10	CTX-M-1	CTX-M-32	AMP, TIC, P, CRM, CFE, FOT, CPD, CAZ, FEP, AZT		

^a^School code: 1, Ángel González; 2, Antanes School; 3, Carmen Conde; 4, León Felipe; 5, Lope de Vega; 6, Manuel Vázquez Montalbán.

^b^Compounds abbreviations: AMP, Ampicillin; TIC, Ticarcillin; P, Piperacillin; MEC, Mecillinam; AMC, Amoxicillin-Clavulanic; FOX, Cefoxitin; CRM, Cerfurixime; CFE, Cefixime; FOT, Cefotaxime; CPD, Cefpodoxime; CAZ, Ceftazidime; FEP, Cefepime; AZT, Aztreonam; AK, Amikacin; GEM, Gentamicin; TOB, Tobramycin; NXN, Norfloxacin; CIP, Ciprofloxacin; LEV, Levofloxacin; NIT, Nitrofurantoin; TRI, Trimethoprim; TRS, Trimethoprim-Sulfamethoxazol. Other compounds tested but with nonsusceptibility for all strains were: P/T, Piperacillin-Tazobactam; FOTC, Cefotaxime-Clavulanic; CAZC, Ceftazidime-Clavulanic; ERT, Ertapenem; IMI, Imipenem; MER, Meropenem; NAL, Nalidixic acid; FOS, Fosfomycin, COL, Colistin, TGC, Tigecycline. Abbreviations in italics indicate intermediate susceptibility.

^c^No clonal complex and ST were assigned because this isolate was identified as the species *Escherichia marmotae.*

Relationships between ST and clonal complex distribution and sociodemographic data collected from the children were assessed, but no statistically significant differences were found for any of the variables considered (Supplementary Table 2).

### 3.4. Distribution of ESBL genes carried by isolates

To further characterize the ESBL carried by each isolate, the *bla* gene was sequenced (Table 2). The *bla* genes coding ESBL detected were *bla*_CTX-M-14_ (29%), *bla*_CTX-M-15_ (21%), *bla*_SHV-12_ (21%), *bla*_CTX-M-27_ (12.5%), *bla*_CTX-M-32_ (12.5%), and *bla*_CTX-M-9_ (4.0%).

**Figure 3:**
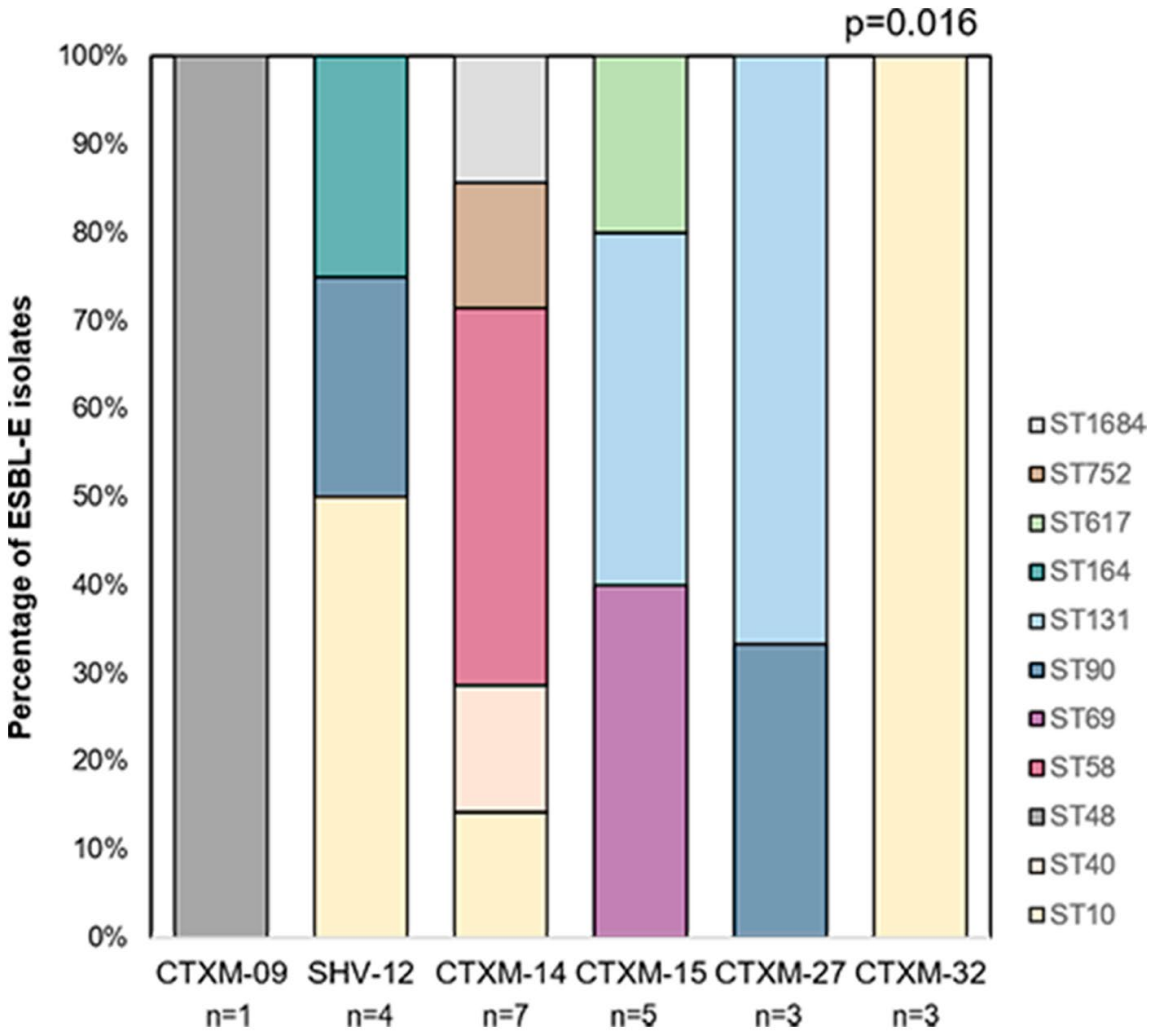
Distribution of ESBL-E isolates ST by *bla* gene allele. Only those isolates for which a ST could be assigned are represented.

No relation between clonal complex and ESBL gene was revealed (Supplementary Table 2). In contrast, ST distribution was significantly different for each ESBL type (*p* = 0.016, Figure 3). Despite the low number of isolates of each *bla* variant, we detected that those of the high risk clone ST131 could feature two different alleles, *bla*_CTX-M-15_ and *bla*_CTX-M-27_. Different allelles were also found in isolates from ST10 (*bla*_CTX-M-14,_ *bla*_CTX-M-32_, and *bla*_SHV-12_) and ST90 (*bla*_CTX-M-27,_ and *bla*_SHV-12_).

### 3.5. Antibiotic resistance profile of ESBL containing isolates

ESBL-E resistance profile was phenotypically assessed by testing susceptibility to 32 clinically relevant antibiotics (Table 2 and Supplementary Table 3). Regarding ß-lactams, all ESBL-E isolates were resistant to ampicillin, ticarcillin and piperacillin and cefotaxime, whereas 88% of isolates were susceptible to amoxicillin-clavulanic. Regarding cephalosporins, all isolates except one were susceptible to cefoxitin (4.1%). In addition, 33% of isolates were susceptible to ceftazidime. All isolates were susceptible to piperacillin-tazobactam and carbapenems.

Concerning quinolones, eight isolates (33%) featured co-resistance to norfloxacin, ciprofloxacin and levofloxacin. Regarding aminoglycosides, two isolates were non-susceptible to amikacin (8%), five isolates were non-susceptible to tobramycin (21%) and, in addition, two of them presented non-susceptibility to gentamicin (8.3%). One strain (4.1%) was non-susceptible to nitrofurantoin, whereas none of the strains were resistant to fosfomycin, colistin, and tigecycline. Finally, 11 isolates (46%) were non-susceptible to both trimethoprim and trimethoprim-sulfamethoxazol.

Altogether, ESBL-E isolates demonstrated non-susceptibility to a range between 9 and 18 antibiotics of clinical use, including ß-lactams, aminoglycosides, and quinolones among others. In the present study, four isolates (17%) were resistant to three or more antimicrobial classes used to treat Enterobacterales infections, according to previous guidelines (Magiorakos et al., 2012).

**Figure 4:**
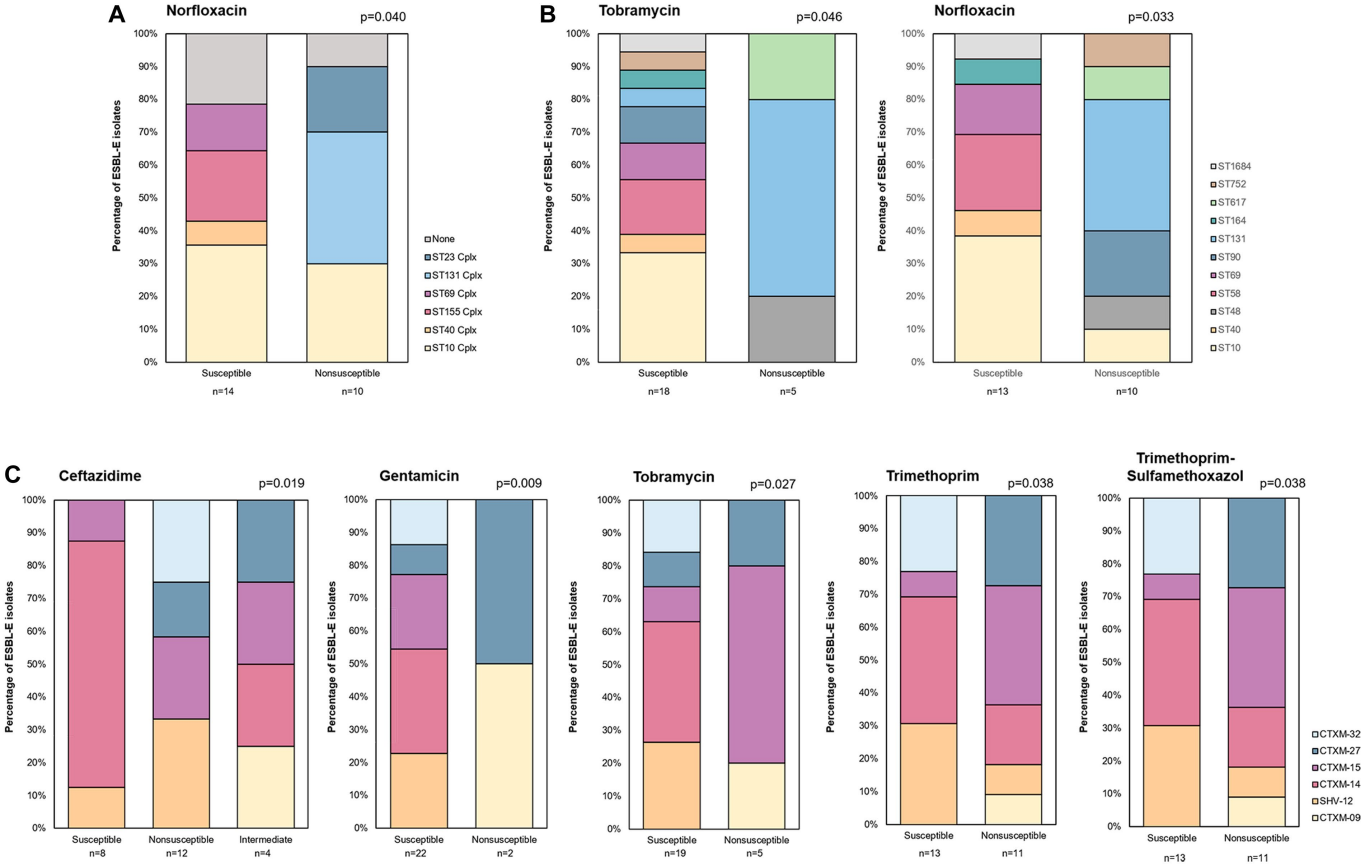
Distribution of ESBL-E isolates by clonal complex **(A)** ST **(B)** and *bla* gene allele **(C)** according to resistance profile to several antibiotics.

Regarding the association of antibiotic resistance with clonal complex, significant differences were observed for norfloxacin (*p* = 0.040) (Supplementary Table 4). Whereas isolates from ST131 Cplx, and ST23 Cplx were all non-susceptible to this antibiotic, isolates of ST40 Cplx, ST155 Cplx and ST69 Cplx were all susceptible (Figure 4A). In line with this, significant differences in ST distribution were observed in relation to resistance to norfloxacin (*p* = 0.033), and tobramycin (*p* = 0.046) (Supplementary Table 4 and Figure 4B). Of note, a higher percentage of isolates non-susceptible to tobramycin was found to belong to ST131. Regarding the association between *bla* allele and resistance profiles, we found differences in prevalence with regard to ceftazidime, gentamicin, tobramycin, trimethoprim and trimethoprim-sulfamethoxazol susceptibility (*p* ≤ 0.038) (Supplementary Table 4). Isolates with *bla*_SHV-12_ and *bla*_CTX-M-14_ and *bla*_CTX-M-32_ were all susceptible to gentamicin and tobramycin whereas differences in ceftazidime, trimethoprim, and trimethoprim-sulfamethoxazol were mainly due to a higher percentage of susceptible strains with *bla*_CTX-M-14_ strains (Figure 4C).

## 4. Discussion

In this study, we provide an updated picture of ESBL-E fecal carriage in healthy children in Spain, which is of relevance because colonized individuals (i) may have a higher risk for developing subsequent infections by these bacteria, (ii) are an important reservoir of ESBL-E, and (iii) may have daily habits associated with the transmission of this group of multidrug resistant bacteria, worth to be identified to prevent ESBL-E dissemination.

The prevalence of ESBL-E fecal carriage in our cohort was 2.6%, and in all cases but one corresponded to *E. coli* species. This is in agreement with previous reports about this species indicating that *E. coli* is among the most prevalent ESBL-E detected in fecal samples in Spain (Valverde et al., 2004; Rodríguez-Baño et al., 2008; Vinué et al., 2009; Garrido et al., 2014; Ríos et al., 2017) and among the most frequent species with antimicrobial resistance in Europe (European Centre for Disease Prevention and Control, 2020). In addition, fecal colonization with *E. coli* producing ESBL has been previously reported in the Spanish community including both children and adults (8 months to 85 years old), with prevalence rates ranging from 3.7 to 67.9% (Valverde et al., 2004; Rodríguez-Baño et al., 2008; Vinué et al., 2009; Fernández-Reyes et al., 2014). In contrast, other studies involving several European countries reported that ESBL-E prevalence in healthy children is below 5.0% (Birgy et al., 2012; Kaarme et al., 2013), which is consistent with our data. Differences in colonization rate between these studies and our analysis can be due to the methodological approach used as well as the cohort engaged. Importantly, the isolation procedure used in this study highlighted the need of including several confirmation steps even when selective medium is used, as we demonstrated that of the 128 presumptive ESBL-E carriers initially identified, only 23 were confirmed. In our hands, over 75% of isolates recovered in the selective medium were not Enterobacterales (not glucose/lactose fermenters after growth on Kligler medium) or *bla* could not be detected by molecular methods (Figure 1). The latter belonged to *Proteus columbae* and *Citrobacter pasteurii* species. False-positive results with chromID ESBL medium were previously reported due to strains overproducing chromosomal cephalosporinase or a chromosomal penicillinase (Réglier-Poupet et al., 2008). In line with this, overproduction of chromosomally encoded AmpC of *Citrobacter* sp., *Enterobacter* sp. and *E. coli* generate resistance to third generation cephalosporin (Meini et al., 2019). In addition, *P. penneri* and *P. vulgaris* can become resistant to cephalosporins as consequence of chromosomally codified Hug A and *Cum* A penicillinases, respectively (Cantón et al., 2006) and species of these genus were detected in the present study.

Although most of the children carried a single ESBL-E, we detected a carrier of two different ESBL-E strains according to PFGE, MLST, and antimicrobial resistance profile. Previous studies have also reported isolation of two different *E. coli* producing ESBL from fecal samples obtained from Spanish children (Fernández-Reyes et al., 2014) and adults (Valverde et al., 2004; Rodríguez-Baño et al., 2008; Garrido et al., 2014).

Among the demographic factors considered in this study, no differences in gender or by age groups were found, neither in relation to the number of siblings which is in line with previous analyses (Fernández-Reyes et al., 2014). Although water pollution has been reported as a major reservoir for ESBL-E worldwide (Woerther et al., 2013), we did not find significant association with the source of drinking water, probably because most of the participants were tap and/or bottled water consumers. Similarly, no association with international travel was detected in our cohort despite being a previously reported risk factor (Sannes et al., 2008), probably due to the low frequency of international travel among the children studied or that these do not include regions with the highest ESBL-E prevalence (i.e., South-East Asia, Western Pacific or Africa) (Bezabih et al., 2021). Living with pets at home has been indicated as protective for ESBL-E acquisition (Rodríguez-Revuelta et al., 2018) but we did not find differences in prevalence in relation to pet ownership, which is in agreement with a previous study (Fernández-Reyes et al., 2014).

We have observed that the ESBL-E isolated in our study have different PFGE profiles, belonging to several STs and clonal complexes. This may suggest independent colonization events and limited transmission between children attending the same primary school. Low clonal relationships among related (Romero et al., 2005; Oteo et al., 2012) and non-related (Valverde et al., 2004; Vinué et al., 2009; Díaz et al., 2010) ESBL-E isolates have been reported in several studies supporting absence of a circulating clone. In our context the most frequent reasons are person-to-person transmission from a relative (Rodríguez-Baño et al., 2008), but this was not evaluated in the present study as households were not included in our cohort of analysis. In addition, we observed that *E. coli* from ST10 and ST131 clonal complexes were the most frequent. Previously, *E. coli* from these clonal complexes have been reported among the most frequent in Spanish cohorts from both community and hospital settings (Oteo et al., 2009; Ríos et al., 2017). In addition, ST131 was identified as a high-risk clone of multiresistance (Mathers et al., 2015). However, in our study ST10 isolates were more frequent than those of ST131 isolates. This fact may suggest a change in the variant of highest incidence or differences in ESBL-E spread patterns between children and adults, and surveillance to confirm this trend is warranted.

In addition, we detected six different *bla* gene alleles in our collection of ESBL-E isolates, mainly from the CTX-M-1 and CTX-M-9 groups but also from the SHV-12. In contrast to previous studies (Romero et al., 2005), none of the isolates had two different ESBLs. Predominance of CTX-M-like enzymes from these two groups has been previously shown at hospital and community settings in Spain (Valverde et al., 2004, 2008; Romero et al., 2005; Rodríguez-Baño et al., 2008; Vinué et al., 2009; Díaz et al., 2010; Garrido et al., 2014; Ríos et al., 2017) and other European countries (Livermore et al., 2007), including studies in children (Oteo et al., 2012; Rodríguez-Revuelta et al., 2018). Of note, *bla*_CTX-M-14_ and the *bla*_SHV-12_ are among the most frequent ESBL-E alleles found in *E.coli* strains in chicken and other animal foods in Spain (Briñas et al., 2003; Ojer-Usoz et al., 2013). With regard to an association of ST and ESBL genes, we observed for most isolates unique combinations of ST and ESBL gene. However, various ESBL genes were found in isolates of ST10, ST90, and ST131. Previously, ST131 has been linked to *bla*_CTX-M-15_ (Mathers et al., 2015). However, our data shows that it can include other ESBL types, which is in agreement with previous studies (Ríos et al., 2017). Concerning ST10, the most frequently detected in this study, we observed that most of them carry *bla*_CTX-M-32_, although other ESBLs were detected. To the best of our knowledge, no previous association between this ST and this allele has been reported in children.

Previous reports indicate that ESBL genes are often associated with genes encoding resistance to other antimicrobials including aminoglycosides, fluoroquinolones, tetracycline and trimethoprim-sulfamethoxazole (Coque et al., 2008; Guimarães et al., 2009; Rodríguez-Revuelta et al., 2018). Notably, a previous study in 8–16-month-old healthy Spanish children evidenced that over 50% of ESBL-E isolates recovered were resistant to three or more antimicrobial classes (Fernández-Reyes et al., 2014), which is higher than figures found among our isolates. In addition, over 20% of isolates were resistant to tetracycline, trimethoprim-sulfamethoxazole, and levofloxacin (Fernández-Reyes et al., 2014), which is in agreement with our results. Association between *bla* alleles with resistance to several antimicrobials was found. This may point to co-transmission of resistance for instance toward plasmids containing several resistance genes. In line with this, the most prevalent ESBL associated to quinolone resistance plasmids were from the CTX-M and SHV families (Briales et al., 2012). Further studies to determine the genetic environment of these elements would be helpful to understand the origin and spread of these mechanisms of multi-resistance.

The number of children enrolled in the study from a 10-year age span represents the main strengths of this study, which provides a robust analysis of ESBL-E prevalence in this cohort. In addition, the molecular characterization of the isolates has allowed an in-depth characterization of the circulating clones and to provide meaningful information about these resistant microorganisms’ dissemination among the community. Nonetheless, the main findings obtained in this retrospective cross-sectional epidemiological study should be contextualized considering some of its limitations: (i) we cannot extrapolate that our data are representative of the whole metropolitan area of Madrid, given the voluntary basis to participate in the study (for the schools and participants) and a bias toward families more concerned about their wellbeing (and thus prone to participate) may have occurred, (ii) questionnaire data may have some recall/recording bias, and (iii) the samples conservation and screening method that we used may have influenced the ESBL-E detection rate obtained.

## 5. Concluding remark

The present study contributes to active surveillance of multidrug resistant bacteria spread, which may be useful for reducing transmission of antimicrobial-resistant bacteria and preventing infection, particularly in this age group. Further studies aimed at establishing if the time of ESBL-E maintenance within the microbiota would aid in deciphering inter-individual resilience. However, future studies to determine the impact of ESBL-E carriage on the microbial community within the gut, whether or not they belong to transient or established microbial community, and if there is transmission of resistance to other gut commensals would be of interest in order to evaluate intra-individual persistence of resistance.

## Data availability statement

The sequences presented in this study were deposited in the GenBank/EMBL/DDBJ database (https://www.ncbi.nlm.nih.gov/genbank/) under the accession numbers ON921220-ON921243 (16S rRNA), OP160325-OP160348 (*adk*), OP160349-OP160372 (*fumC*), OP160373-OP160396 (*gyrB*), OP160397-OP160420 (*icd*), OP160421-OP160444 (*mdh*), OP160445-OP160468 (*purA*), and OP160469-OP160492 (*recA*).

## Ethics statement

Written informed consent to participate in the study was signed prior to recruitment by the participants’ legal guardian/next of kin of participating schoolchildren, in accordance with the Declaration of Helsinki. Socio-demographic, epidemiological data and stool samples were sent codified to and are published in an aggregated form to assure anonymity of participants. All study procedures, informed consent forms, and epidemiological questionnaires were reviewed and approved by Ethics Committee of the Health Institute Carlos III under the reference number CEI PI 17_2017-v3 on 17th September 2018.

## Author contributions

ML-S performed ESBL-E isolation, phenotype confirmation, MLST, antibiotic resistance profile, data analysis, drafted the manuscript, and approved final version. ZM performed *bla* gene identification and revised the final version of the manuscript. AM, PK, and AD performed samples and data recruitment and revised the final version of the manuscript. RC assisted in ESBL-E isolation and revised the final version of the manuscript. SS performed PFGE and data analysis and revised the final version of the manuscript. AÁ assisted in MLST analysis and revised the final version of the manuscript. NL assisted in PFGE and *bla* gene identification and revised the final version of the manuscript. JO-I conceived the study, revised the manuscript, and granted funding. DC conceived and supervised the study, granted ethics committee approval, revised the manuscript, and granted funding. MM conceived and supervised the study, granted ethics committee approval, drafted the manuscript, and approved final version. All authors contributed to the article and approved the submitted version.

## Funding

ML-S was supported by the Sara Borrell Program of the Instituto de Salud Carlos III (ISCIII) (CD17CIII/00017). ZM was supported by the Río Hortega Program of the ISCIII. AÁ was supported by the Garantía Juvenil Program of the Comunidad Autonoma de Madrid. SS was supported by the Miguel Servet program of ISCIII (CPII18CIII/00005). This study was funded by the ISCIII, Ministry of Economy and Competitiveness (Spain), under projects PI16CIII/00024, PI18CIII/00030, MPY 380/18, and MPY 516/19.

## Conflict of interest

MM was founder and stockholder of the biotechnology spin-off company Vaxdyn, which develops vaccines for infections caused by MDR bacteria. Vaxdyn had no role in the elaboration of this manuscript.

The remaining authors declare that the research was conducted in the absence of any commercial or financial relationships that could be construed as a potential conflict of interest.

## Publisher’s note

All claims expressed in this article are solely those of the authors and do not necessarily represent those of their affiliated organizations, or those of the publisher, the editors and the reviewers. Any product that may be evaluated in this article, or claim that may be made by its manufacturer, is not guaranteed or endorsed by the publisher.

## Footnotes

^1^ https://www.cdc.gov/pulsenet

^2^ https://www.cdc.gov/pulsenet/pdf/ecoli-shigella-salmonella-pfge-protocol-508c.pdf
