# Supplementary material for: Fecal carriage of extended-spectrum beta-lactamase-producing Enterobacterales in healthy Spanish schoolchildren
Source: Front Microbiol. 2023 Jun 9;14:1035291. doi: 10.3389/fmicb.2023.1035291 (PMC10288999; doi:10.3389/fmicb.2023.1035291)
Supplement: Supplementary file 1 [file Data_Sheet_1.docx]

IPQ4: We noticed that changes were made to the DAS statements. Please check if these changes are fine.

Old version: The datasets presented in this study can be found in online repositories. The names of the repository/repositories and accession number(s) can be found: https://www.ncbi.nlm.nih.gov/genbank/, ON921220-ON921243, https://www.ncbi.nlm.nih.gov/genbank/, OP160325-OP160348, https://www.ncbi.nlm.nih.gov/genbank/, OP160349-OP160372, https://www.ncbi.nlm.nih.gov/genbank/, OP160373-OP160396, https://www.ncbi.nlm.nih.gov/genbank/, OP160397-OP160420, https://www.ncbi.nlm.nih.gov/genbank/, OP160421-OP160444, https://www.ncbi.nlm.nih.gov/genbank/, OP160445-OP160468, https://www.ncbi.nlm.nih.gov/genbank/, OP160469-OP160492.

Author Responses: The sequences presented in this study were deposited in the GenBank/EMBL/DDBJ database (https://www.ncbi.nlm.nih.gov/genbank/) under the accession numbers ON921220-ON921243 (16S rRNA), OP160325-OP160348 (*adk*), OP160349-OP160372 (*fumC*), OP160373-OP160396 (*gyrB*), OP160397-OP160420 (*icd*), OP160421-OP160444 (*mdh*), OP160445-OP160468 (*purA*), and OP160469-OP160492 (*recA*). The raw data supporting the conclusions of this article will be made available by the authors upon request, without undue reservation.

IPQ4: We noticed that changes were made to the Ethics statements. Please check if these changes are fine.

Old version: The studies involving human participants were reviewed and approved by Ethics Committee of the Health Institute Carlos III under the reference number CEI PI 17_2017-v3 on 17th September 2018. Written informed consent to participate in this study was provided by the participants’ legal guardian/next of kin.

Author Responses: Written informed consent to participate in the study was signed prior to recruitment from by the participants’ legal guardian/next of kin of participating schoolchildren, in accordance with the Declaration of Helsinki. Socio-demographic, epidemiological data and stool samples were sent codified to and are published in an aggregated form to assure anonymity of participants. All study procedures, informed consent forms, and epidemiological questionnaires were reviewed and approved by Ethics Committee of the Health Institute Carlos III under the reference number CEI PI 17_2017-v3 on 17th September 2018.
